# Prospective phase II trial of everolimus in PIK3CA amplification/mutation and/or PTEN loss patients with advanced solid tumors refractory to standard therapy

**DOI:** 10.1186/s12885-017-3196-6

**Published:** 2017-03-23

**Authors:** Seung Tae Kim, Jeeyun Lee, Se Hoon Park, Joon Oh Park, Young Suk Park, Won Ki Kang, Ho Yeong Lim

**Affiliations:** Department of Medicine, Division of Hematology-Oncology, Samsung Medical Center, Sungkyunkwan University School of Medicine, 81 Irwon-ro Gangnam-gu, Seoul, 135-710 Korea

**Keywords:** PIK3CA amplification/mutation, PTEN loss, everolimus

## Abstract

**Background:**

We designed a single-arm, open-label phase II trial of everolimus in PIK3CA amplification/mutation and/or PTEN loss patients with advanced solid tumors refractory to standard therapy (#NCT02449538).

**Methods:**

Everolimus was administered orally at a daily dose of 10 mg continuously (28-day cycles). Treatment was continued until progression of the disease or intolerable toxicity was observed. Based on Simon’s two-stage optimal design, 10 patients were treated with everolimus during the first stage.

**Results:**

The median age of the patients was 55.5 years (range, 42–72), and the median Eastern Cooperative Oncology Group (ECOG) performance status (PS) was 2 (range, 1–2). Most of the patients (50.0%) had gastric cancer (GC) as the site of their primary tumor followed by colorectal cancer (CRC), pancreatic cancer, and cholangiocarcinoma. Patients received everolimus as a third-line (3 patients), fourth-line (4 patients), fifth-line (1 patient) or sixth-line (2 patients) treatment. Complete or partial responses were not observed in any of the patients. Four patients showed stable disease, resulting in a disease control rate of 40%. The median PFS was 1.6 months (95% CI, 0.8–2.4 months). Grade 3 or greater hematologic/non-hematologic toxicity was not observed. Grade 2 diarrhea and stomatitis were reported in one patient each. There were no treatment-related deaths. There was less than one response out of the 10 initial patients during the first stage, and the study did not progress to the second stage.

**Conclusions:**

The study did not meet its primary objective of demonstrating the anti-tumor activity of everolimus in PIK3CA amplification/mutation and/or PTEN loss patients with advanced solid tumors refractory to standard therapy. Further investigation using other genomic candidates and new-generation mTOR inhibitors is warranted in patients with treatment-refractory cancer.

**Trial registration:**

#NCT02449538, April 2015.

## Background

Clinical development process of molecularly targeted agents for cancer and is similar to that of cytotoxic agents are pretty similar, targeting tumor location and histology [1–3]. Irrespective of different tumor types and histology, Mmost molecular alterations exist irrespective of different tumor types and histologies, although the incidence can varyies [4]. This observation challenges existing drug development strategies for molecularly targeted agents and raises the possibility of a shift towards histology-agnostic molecularly-based treatment [5].

The mechanisms of cancer are marked by complex aberrations in active and critical cellular signaling pathways involved in tumorigenesis [6]. The phosphoinositide 3-kinase (PI3K)-v akt murine thymoma viral oncogene homolog (AKT)-mechanistic target of rapamycin (mTOR) signaling cascade is one of the most important intracellular pathways that is frequently activated in diverse cancers [7, 8]. In many types of tumors, the activation of the PI3K-AKT-mTOR pathway has been known as the relation to tumorigenesis, cancer progression and the acquired resistance to various anti-neoplastic agents [7, 9]. mTOR is an evolutionarily conserved serine/threonine kinase which acts downstream of the PI3K pathways. Thus, inhibition of the mTOR pathway represents a novel therapeutic strategy in the treatment of various cancers [10–13]. Everolimus, an mTOR inhibitor, demonstrates antiproliferative activity through the inhibition of the PI3K-AKT-mTOR pathway and also has antiangiogenic effects [14, 15]. Everolimus has shown antitumor activity in various types of tumors, but, it activity has limited in only a subset of cancer patients [11, 12, 16, 17]. However, there have not been predictive biomarkers for everolimus, until now. Therefore, novel biomarkers are needed to identify patients who would receive the most benefit from everolimus treatment. Recently, in several studies, PIK3CA/PTEN genomic aberrations have been suggested to be strong predictors of everolimus sensitivity [18–21]. PIK3CA amplifications and mutations have been implicated in pathway activation and sensitivity to mTOR inhibitors. Some preclinical models have further shown that PTEN-deficient tumors present an enhanced sensitivity to mTOR inhibitors because of the sustained activation of PI3K-AKT signaling [22, 23]. These findings have enabled researchers to apply mTOR inhibitors in many tumor-types with specific genomic aberrations irrespective of tumor histology and location.

We designed a single-arm, open-label phase II trial of everolimus in PIK3CA amplification/mutation and/or PTEN loss patients with advanced solid tumors refractory to standard therapy (#NCT02449538).

## Methods

### Eligibility

Patients were eligible if they had a histologically-confirmed solid cancer with PTEN loss and/or PIK3CA amplification/mutation. The additional case inclusion criteria were as follows: (1) age over 18; (2) an Eastern Cooperative Oncology Group (ECOG) performance status of 0 or 1; (3) adequate bone marrow, liver, and renal function; (4) life expectancy of at least 3 months. Patients who have an acute active infection were not included in this study. Patients who have any prior history of another malignancy within 5 years of entry into the study, apart from nonmelanoma skin cancer or carcinoma in situ of the uterine cervix, were precluded participation in this study. In addition, we did not include any patients with known brain metastasis and concurrent uncontrolled hypertension, symptomatic congestive heart failure, unstable angina pectoris, significant cardiac arrhythmia, or severe psychiatric illness in this study. All patients provided written informed consent according to the guidelines provided by the institutional review board and all procedures were carried out according to guidelines from the Declaration of Helsinki. The Institutional Review Board at Samsung Medical Center approved the protocol.

PTEN loss was confirmed by immunohistochemistry (IHC) test. Immunohistochemical staining was performed on 3 um thick sections from each case on a BOND-MAX autostainer (Leica, Melbourne, Australia) using BondTM Polymer refine detection, DS9800 (Vision Biosystems, Melbourne, Australia) after retrieval with T/E buffer. We used primary antibodies to PTEN (1:100, Cellsignaling, #9559). To evaluate the loss of PTEN expression, positive staining of blood vessels and stromal cells were used as an internal positive control. We classified the PTEN expression based on a four grading system: loss, weak positive expression, moderate positive expression and strong positive expression with the later three categories being lamped as the expression. PIK3CA amplification/mutation was detected by targeted deep sequencing by CancerSCAN. Briefly, extracted genomic DNA was sheared to 150–200 bp using Covaris S220 (Covaris, Woburn, MA) and targeted genes were captured using custom panel capture library (Agilent Technologies, Santa Clara, CA) for 2.5 Mb of exonic regions for Illumina Paired-End Sequencing Library kit. We performed DNA sequencing of 100 or 101-bp paired-end reads using the Illumina HiSeq 2500 sequencer (Illumina, San Diego, CA).

### Chemotherapy

Everolimus was administered orally at a daily dose of 10 mg continuously (28 day cycles). Treatment was continued from day 1 until progression of the disease, unacceptable toxicity, or the patient’s request. Thereafter, the patients were followed up. Dose modification was allowed in patients unable to tolerate the dosing schedule defined by the protocol. If the toxicity was tolerable for the patient, the starting dose was maintained. Dose reductions for toxicity were as follows: dose level 1, 5 mg daily; dose level 2, 5 mg every other day.

### Assessment of efficacy and toxicity

Tumor response was routinely assessed with CT scan every two cycles until the progression of disease. The response was evaluated using a method identical to that used in Response Evaluation Criteria in Solid Tumors (RECIST) criteria, version 1.1. Toxicities were assessed according to the National Cancer Institute Common Terminology Criteria for Adverse Events (NCI-CTCAE version 4.0).

### Statistical analysis

According to Simon’s two-stage optimal design, a sample size of 23 was required to accept the hypothesis that the true RR is greater than 25% with 80% power, and to reject the hypothesis that the RR is less than 5% with 5% significance. In the first stage, if there was less than one response out of the initial 10 patients, the study would be terminated. Although the target number of patients was 23, we planned to recruit 10% more than the target number of patients due to expected dropout. Descriptive statistics were reported as proportions and medians. Kaplan–Meier estimates were used in the analysis of all time-to event variables, and the 95% confidence interval (CI) for the median time to event was computed.

## Results

### Patients

We screened total 82 patients with solid cancer between April 2015 and December 2015. Among 82 patients, 29 patients had PIK3CA alterations or PTEN loss and then 10 patients were enrolled onto this study. These 10 patients were treated with everolimus in the first stage of Simon’s two-stage optimal design. The median age of the patients was 55.5 years (range, 42–72), and there were 7 male and 3 female patients. Around half (50.0%) of the patients had gastric cancer (GC) as the site of the primary tumor followed by colorectal cancer (CRC), pancreatic cancer and cholangiocarcinoma. Table 1 shows the baseline characteristics. The tumor samples of nine patients were confirmed to show PTEN loss by IHC and one GC with the PIK3CA mutation was found by target sequencing (Table 2).

**Table 1:** Baseline Characteristics of patients in this study

Characteristics	No, of patients	Percent
Age		
Median (Range)	55.5 (42.0–72.0)	
ECOG performance status		
Median (Range)	2 (1–2)	
Gender		
Male	7	70%
Female	3	30%
Race		
Asian	10	100%
Country		
South Korea	10	100%
Disease Type		
Gastric cancer	5	50%
Colorectal cancer	3	30%
Pancreas cancer	1	10%
Cholangiocarcinoma	1	10%
Disease Status		
Metastatic	9	90%
Recurrent	1	10%
Pathologic Type		
Well differentiated	1	10%
Moderate differentiated	5	50%
Poorly differentiated	4	40%
Metastatic sites		
Liver	4	40%
Lymph node	7	70%
Peritoneum	3	30%
No. of metastatic sites		
≤ 1	7	70%
2 ≤	3	30%
Prior lines of therapy		
2	3	30%
3	4	40%
4	1	10%
5	2	20%

**Table 2:** The status of molecular markers in enrolled patients

Disease types	Molecular markers	
	PTEN loss	PIK3CA mutation/amplification
Gasric cancer	4	1
Colorectal cancer	3	-
Pancreastic cancer	1	-
Cholangiocarcinoma	1	-

### Response and survival

**Fig. 1:**
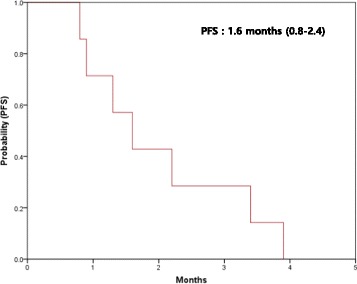
Progression free survival of enrolled patients

Response outcomes are listed in Table 3. Response evaluation was conducted in the intent-to-treat population. A complete or partial response was not observed in any of the patients. Four patients showed stable disease and 4 patients showed disease progression. The disease stabilization rate was achieved in 40% of all patients. The evaluation of treatment-response was not available in two patients. All two patients were lost to follow-up before the first response evaluation. All 10 patients were included in survival analysis on an intent-to-treat basis. The median PFS was 1.6 months (95% CI, 0.8–2.4 months) (Fig. 1). Treatment was discontinued due to disease progression in all enrolled patients. There was less than one response out of the initial 10 patients during the first stage, and the study did not continue into the second stage.

**Table 3:** Treatment response of enrolled patients

Response	No. of patients	Percent
Complete response	-	-
Partial response	0	0%
Stable disease	4	40%
Progressive disease	4	40%
Not available	2	20%
Overall response rate	0%	
Disease control rate	30%

### Delivery of the drug and toxicity

The patients received a median of 1.7 (range, 1–4) cycles. Dose reduction or treatment delay was not required in the enrolled patients. Grade 3 or greater hematologic/non-hematologic toxicity was not observed. Grade 2 diarrhea and stomatitis were reported in one patient each. There were no treatment-related deaths.

## Discussion

mTOR is a key down-stream protein kinase of the PI3K-AKT signaling pathway, and everolimus is a novel macrolide derivative of rapamycin that inhibits mTOR, thereby preventing phosphorylation of its down-stream molecules. Its promising antitumor effect has been reported in various tumor types such as renal cell carcinoma, biliary tract cancer, gastric cancer, and neuroendocrine tumor [10, 11, 13, 17]. PIK3CA/PTEN genomic aberrations have been suggested to be strong predictors of everolimus sensitivity. In the present study, we sought to investigate the anti-tumor activity of everolimus in solid tumors with specific genotypes such as PIK3CA amplification/mutation and/or PTEN loss. However, this study did not meet the primary end point of the first stage of Simon’s two stage design and was terminated without proceeding to the second stage. To proceed to the stage, at least one patient with CR or PR was needed at the end of the first stage of the study. Although there were four patients with SD among the 10 patients enrolled in the first stage, there was no CR or PR. Inconsistent findings among clinical trials for everolimus may be caused by heterogeneous patients’ population such as different tumor-types, different races and etc.

mTOR is composed of two distinct protein complexes, mTORC1 and mTORC2, that act on different levels of the pathway [24, 25]. Unlike the mTORC1 complex, mTORC2 positively regulates cell survival and proliferation on different signaling levels, mainly by phosphorylation as well as through a serum and glucocorticoid inducible kinase [26]. Everolimus usually acts as an allosteric inhibitor of the mTORC1 complex through interaction with FK-binding protein 12 [27]. Thus, everolimus is generally thought to have only weak activity against the mTORC2 complex, which can lead to AKT activation [28]. This might have affected the outcome of this study. Although this study selected its subpopulation using specific genotypes that are known as predictive markers for everolimus, mTOR signaling via the mTORC2 complex could not be blocked. Newly developed mTOR kinase blockades are expected to provide a more robust inhibition of mTOR signaling via suppression of both mTORC1 and mTORC2 complexes. These agents are now in preclinical and clinical trials and include TAK228/INK128, AZD8055, and AZD2014 [29–31]. We have also conducted phase II trial using AZD2014 in gastric cancer (#NCT02449655).

Patients who achieved disease control with everolimus had GC (*n* = 2), CRC (*n* = 1), and chonlangiocarcinoma (*n* = 1). All of these patients had tumors with PTEN loss and did not have other genomic aberrations such as HER2 amplification, RAS mutation, and BRAF mutation that might affect the PI3K-AKT-mTOR signaling. The mTOR signaling pathway is linked to multiple levels of feedbacks and diverse signal crosstalk. Although this study used biomarker-driven patient selection, PTEN loss and PIK3CA amplification/mutation alone were not sufficient for predicting the anti-tumor activity of everolimus. Janku et al. reported various molecular aberration including PIK3CA, PTEN, KRAS, NRAS, and BRAF in 1656 patients with PI3K/AKT/mTOR inhibitors [32]. Thus, more comprehensive molecular analysis will be helpful to fully realize the potential of personalized medicine using mTOR inhibitors including everolimus.

This study revealed that everolimus was tolerable in heavily pretreated patients. Grade 3 or greater hematologic and non-hematologic toxicity was not observed. Grade 2 diarrhea and stomatitis was reported in one patient each. The 10 enrolled patients had already received many types of cytotoxic chemotherapy before everolimus and most had an ECOG PS of 2 (*n* = 9). Considering these characteristics, everolimus was tolerable and safe.

## Conclusion

The study did not meet its primary objective of demonstrating the anti-tumor activity of everolimus in PIK3CA amplification/mutation and/or PTEN loss patients with advanced solid tumors refractory to standard therapy. A greater understanding of the feedback mechanism and crosstalk linked to the mTOR signaling-pathway is needed. Further investigation using other potential genomic candidates and next-generation mTOR inhibitors is warranted in patients with refractory cancer.

## Abbreviations

AKT: v akt murine thymoma viral oncogene homolog
ANC: Absolute neutrophil count
AST/ALT: Aspartate aminotransferase/alanine aminotransferase
CRC: Colorectal cancer
ECOG: Eastern Cooperative Oncology Group
GC: Gastric cancer
IHC: Immunohistochemistry
mTOR: Mechanistic target of rapamycin
NCI-CTC: National Cancer Institute common toxicity criteria
PI3K: The phosphoinositide 3-kinase
PS: Performance Status
